# Minimal Intervention for Controlling Nosocomial Transmission of Methicillin-Resistant *Staphylococcus aureus* in Resource Limited Setting with High Endemicity

**DOI:** 10.1371/journal.pone.0100493

**Published:** 2014-06-19

**Authors:** Vincent Chi-Chung Cheng, Josepha Wai-Ming Tai, Pui-Hing Chau, Jonathan Hon-Kwan Chen, Mei-Kum Yan, Simon Yung-Chun So, Kelvin Kai-Wang To, Jasper Fuk-Woo Chan, Sally Cheuk-Ying Wong, Pak-Leung Ho, Kwok-Yung Yuen

**Affiliations:** 1 Department of Microbiology, Queen Mary Hospital, Hong Kong Special Administrative Region, China; 2 Infection Control Team, Queen Mary Hospital, Hong Kong Special Administrative Region, China; 3 School of Nursing, The University of Hong Kong, Hong Kong Special Administrative Region, China; 4 Carol Yu Centre for Infection, The University of Hong Kong, Hong Kong Special Administrative Region, China; Hospital de São Francisco Xavier, CHLO, Faculty of Medical Sciences, New University of Lisbon, Portugal

## Abstract

**Objective:**

To control nosocomial transmission of methicillin-resistant *Staphylococcus aureus* (MRSA) in resource-limited healthcare setting with high endemicity.

**Methods:**

Three phases of infection control interventions were implemented in a University-affiliated hospital between 1-January-2004 and 31-December-2012. The first phase of baseline period, defined as the first 48-months of the study period, when all MRSA patients were managed with standard precautions, followed by a second phase of 24-months, when a hospital-wide hand hygiene campaign was launched. In the third phase of 36-months, contact precautions in open cubicle, use of dedicated medical items, and 2% chlorhexidine gluconate daily bathing for MRSA-positive patients were implemented while hand hygiene campaign was continued. The changes in the incidence rates of hospital-acquired MRSA-per-1000-patient admissions, per-1000-patient-days, and per-1000-MRSA-positive-days were analyzed using segmented Poisson regression (an interrupted time series model). Usage density of broad-spectrum antibiotics was monitored.

**Results:**

During the study period, 4256 MRSA-positive patients were newly diagnosed, of which 1589 (37.3%) were hospital-acquired. The reduction of hospital-acquired MRSA per 1000-patient admissions, per 1000-patient-days, and per 1000-MRSA-positive-days from phase 1 to 2 was 36.3% (p<0.001), 30.4% (p<0.001), and 19.6% (p = 0.040), while the reduction of hospital-acquired MRSA per 1000-patient admissions, per 1000-patient-days, and per 1000-MRSA-positive-days from phase 2 to 3 was 27.4% (p<0.001), 24.1% (p<0.001), and 21.9% (p = 0.041) respectively. This reduction is sustained despite that the usage density of broad-spectrum antibiotics has increased from 132.02 (phase 1) to 168.99 per 1000 patient-days (phase 3).

**Conclusions:**

Nosocomial transmission of MRSA can be reduced with hand hygiene campaign, contact precautions in open cubicle, and 2% chlorhexidine gluconate daily bathing for MRSA-positive despite an increasing consumption of broad-spectrum antibiotics.

## Introduction

The control of nosocomial transmission of methicillin-resistant *Staphylococcus aureus* (MRSA) in endemic areas of Asia, Europe, and North America [1], [2], [3], [4] has demonstrated various degree of success with the implementation of active surveillance culture, isolation of MRSA-colonized patients, hand hygiene practice, environmental cleanliness, targeted or universal decolonization, and antimicrobial stewardship program. Remarkably, a decreasing trend in MRSA bacteremia has been seen since 2006 in UK, following a government initiative to establish a mandatory surveillance and a target to reduce MRSA bacteremia rates over a 3-year period [5], facilitated by the corresponding optimization of administrative support, infrastructural change and resource allocation [1].

However, the control of MRSA in highly endemic healthcare setting is more challenging where resources are limited and isolation facilities are scarce. In Hong Kong, existing hospital buildings have outdated designs with limited number of isolation rooms. Hence, patients colonized or infected with vancomycin-resistant *Enterococci* and carbapenem-resistant *Enterobacteriaceae*, agents not yet endemic in our healthcare setting, are prioritized to be isolated in these rooms [6], [7]. Meanwhile, our hospital-wide MRSA control program has to depend on the use of hand hygiene campaign [8], which has been associated with reduction of MRSA transmission [9], and contact precautions without single room isolation facility.

## Materials and Methods

A retrospective study was conducted to determine the nosocomial transmission of MRSA in Queen Mary Hospital, a 1600-bed tertiary referral university-affiliated teaching hospital, between 1 January 2004 and 31 December 2012. Our study period was divided into three phases: (i) phase 1– baseline observation period from 1 January 2004 to 31 December 2007; (ii) phase 2– launch of the first intervention (hospital-wide hand hygiene campaign using alcohol-based hand rub) from 1 January 2008 to 31 December 2009), and (iii) phase 3– launch of the second intervention (stepwise implementation of contact precautions in open cubicle with use of dedicated medical items including blood pressure cuff, stethoscope, oximeter, tourniquet, and thermometer, and 2% chlorhexidine gluconate daily bath till discharge for MRSA-positive patients) from 1 January 2010 to 31 December 2012. Before the launch of hand hygiene practice campaign in phase 2 and 3, baseline hand hygiene data was collected in 2007. Hand hygiene audit was continued throughout phase 2 and 3. Briefly, our infection control nurses performed hand hygiene audit in each ward at least once a month, monitoring at least 200 hand hygiene opportunities in each ward per year. Among 200 hand hygiene opportunities, 130 were of nurses, 40 of medical doctors, 20 of healthcare assistants, and 10 of allied health professional. Active surveillance of MRSA was not routinely performed due to limited resources. Clinical specimens were collected for microbiological investigation when clinically indicated. MRSA was identified according to our previous laboratory protocol [10], [11]. This study was approved by the Institutional Review Board at Queen Mary Hospital. Written informed consent was not obtained because the present study was a retrospective analysis of a hospital infection control program. Patient records and information were anonymized and de-identified prior to analysis.

### Epidemiology of MRSA

The collection method of epidemiological information of patients with laboratory culture of MRSA remained unchanged during the entire study period. Briefly, the patient admission number and total patient-days were obtained from the computer system of the hospital record office. The data of clinical isolation of MRSA was obtained from the laboratory information system. New cases of hospital-acquired and community-acquired MRSA were defined as the first isolate of MRSA identified after 48 hours and within 48 hours of hospitalization, respectively, without preceding culture positive of MRSA in the past 12 months. Incidence of hospital-acquired MRSA per 1000 patient admissions, per 1000 patient-days, and per 1000 MRSA-positive-days were defined by dividing the number of new cases of hospital-acquired MRSA by the numbers of patient admissions, patient-days, and MRSA-positive-days respectively, in the period concerned. Incidence density of community-acquired MRSA per 1000 patient admissions was calculated in similar manner for comparison. The number of MRSA-positive-days was defined as the number of the total MRSA patient-days in the hospital, measuring from the time interval between collection of clinical culture and patient discharge, in patients with community-acquired and hospital-acquired MRSA.

### Infection Control Program for MRSA

New cases of MRSA were identified by the infection control team via communication with the microbiology laboratory as described previously [12]. Except for the intensive care units [11], [13], all patients with MRSA colonization or infection were managed with standard precautions and cohort nursing in open cubicle in the baseline period due to limited number of isolation rooms (phase 1). During phase 2 and 3, hospital-wide hand hygiene practice using alcohol-based hand rub was promoted. In phase 3, contact precautions were also enforced in the management of MRSA-positive in open cubicles with the provision of dedicated medical items. In addition, daily bathing with 2% chlorhexidine gluconate was provided for all MRSA-positive patients in the later stage in phase 3. Decolonization therapy with intra-nasal mupirocin was given to high risk patients undergoing major surgical interventions such as liver transplantation and cardiac surgery. Outbreak investigation for MRSA was performed when the number of hospital-acquired MRSA cases exceeded 2 standard deviations (SD) of the mean value over the past 12 months in a particular ward. The environmental cleaning protocol remained unchanged throughout the study period, and compliance of hand hygiene was audited regularly [14]. The consumption trend of broad-spectrum antibiotics, expressed as defined daily dose per 1000 patient-days, were obtained from the hospital information system [15].

### Statistical Analysis

Overall patterns of changes in the incidence density of hospital-acquired MRSA (per 1000 patient admissions, per 1000 patient-days, and per 1000 MRSA-positive-days), with respect to the implementation of the infection control interventions, were analyzed using segmented Poisson regression. This model, also known as the interrupted time series model, had been widely used to evaluate interventions related to MRSA incidences [13], [16], [17]. Pattern of changes in the incidence density of community-acquired MRSA per 1000 patient admissions was also examined using segmented Poisson regression for comparison. The full model under investigation was:

ln (λ) = β_0_+ β_1_(I_1_)+β_2_(I_2_)+β_3_(T)+β_4_(T_1_)+β_5_(T_2_), where λ denotes monthly incidence rate, I_1_ denotes hand hygiene practice intervention status (before implementation = 0; after implementation = 1), I_2_ denotes contact precautions intervention status (before implementation = 0; after implementation = 1), T denotes time in months, T_1_ denotes months since implementation of hygiene practice (before implementation = 0), and T_2_ denotes months since implementation of contact precautions (before implementation = 0). Significant factors were selected by stepwise selection procedure. A significance level of 0.05 was adopted. Computation was performed using R Version 3.0.2.

## Results

### Epidemiology of MRSA

A total of 4256 MRSA-positive patients were newly identified during our study period: 2416 (57%) male and 1840 (43%) female, with a median age of 76 years (ranged 1–111 years), resulting in 95,748 MRSA-positive-days. 1731 (40.7%) of 4256 MRSA-positive patients were identified during their hospitalization in the medical unit, while 1076 (25.3%) and 624 (14.7%) were identified in the surgical and orthopedic unit respectively. The remaining 825 (19.3%) MRSA-positive patients were identified in other clinical specialties. Evaluation of the first MRSA-positive clinical specimen received per patient included wound swabs (deep wound, superficial wound, and ulcer), constituted 1380 (32.4%) of 4256 specimens, followed by 1302 (30.6%) respiratory specimens (sputum, tracheal aspirates, bronchoalveolar lavage), 469 (11.0%) urinary specimens (mid-stream urine, catheterized urine, suprapubic catheterization, nephrostomy drain), 365 (8.6%) sterile body fluid or pus, and 280 (6.6%) blood cultures. The epidemiological characteristics of MRSA-positive patients in the 3 phases of intervention were illustrated in Table 1.

**Table 1:** The epidemiological characteristics of MRSA in 3 different phases of intervention.

	Phase 1 (1 Jan 2004to 31 Dec 2007)	Phase 2 (1 Jan 2008to 31 Dec 2009)	Phase 3 (1 Jan 2010to 31 Dec 2012)
Total number of MRSA	1990	877	1389
Total number of hospital-acquired MRSA	890	315	384
Mean number of hospital-acquired MRSA per month	18.5	13.1	10.7
Total number of community-acquired MRSA	1,100	562	1,005
Mean number of community-acquired MRSA per month	22.9	23.4	27.9
Number of MRSA in the major clinical specialties			
Medical unit (hospital- vs community-acquired)	242 vs 481	78 vs 325	108 vs 497
Surgical unit (hospital- vs community-acquired)	327 vs 215	114 vs 85	146 vs 189
Orthopedic unit (hospital- vs community-acquired)	91 vs 193	42 vs 70	55 vs 173
Distribution of MRSA in the major specimen types			
Wound specimens (hospital- vs community-acquired)^a^	206 vs 383	86 vs 209	104 vs 392
Respiratory specimens (hospital- vscommunity-acquired)^b^	361 vs 241	125 vs 133	188 vs 254
Urine specimens (hospital- vs community-acquired)^c^	82 vs 166	32 vs 74	18 vs 97
Sterile body fluid (hospital- vs community-acquired)^d^	38 vs 78	25 vs 57	22 vs 145
Blood culture (hospital- vs community-acquired)	56 vs 91	25 vs 40	21 vs 47
Total number of admissions	414,726	230,474	387,136
Mean number of admissions per month	8,640	9,603	10,754
Total number of patient-days	1,586,799	807,159	1,295,828
Mean number of patient-days per month	33,058	33,632	35,995
Total number of MRSA-positive-days	48,297	16,901	30,550
Incidence densities of MRSA			
Hospital-acquired MRSA per 1000 admissions	2.146	1.367	0.992
Hospital-acquired MRSA per 1000 patient-days	0.561	0.390	0.296
Hospital-acquired MRSA per 1000 MRSA-positive-days	18.428	18.638	12.570
Community-acquired MRSA per 1000 admissions	2.652	2.438	2.596

Note. Phase 1: baseline observation period; phase 2: launch of the first intervention - hand hygiene campaign; phase 3: continuation of phase 2 plus launch of the second intervention - contact precautions.

a wound specimens included deep wound, superficial wound, and ulcer swabs.

b respiratory specimens included sputum, tracheal aspirates, bronchoalveolar lavage;

c urine specimens included mid-stream urine, catheterized urine, suprapubic catheterization, nephrostomy drain urine;

d sterile body fluid included pus.

**Figure 1:**
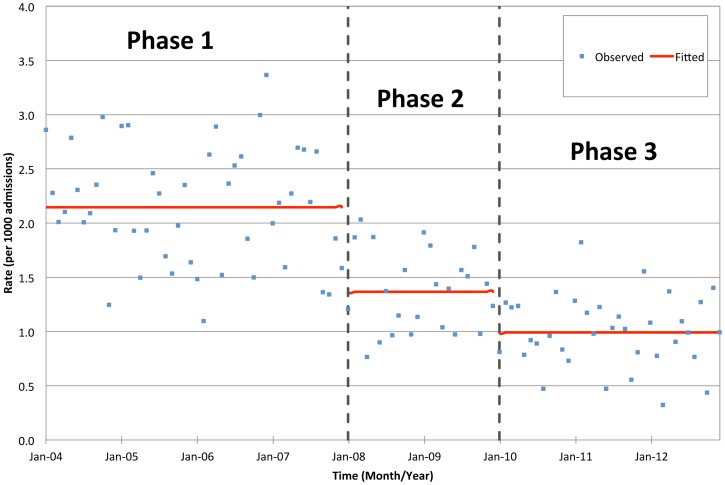
Incidence rate of hospital-acquired MRSA per 1000 admissions in the three phases of intervention.

**Figure 2:**
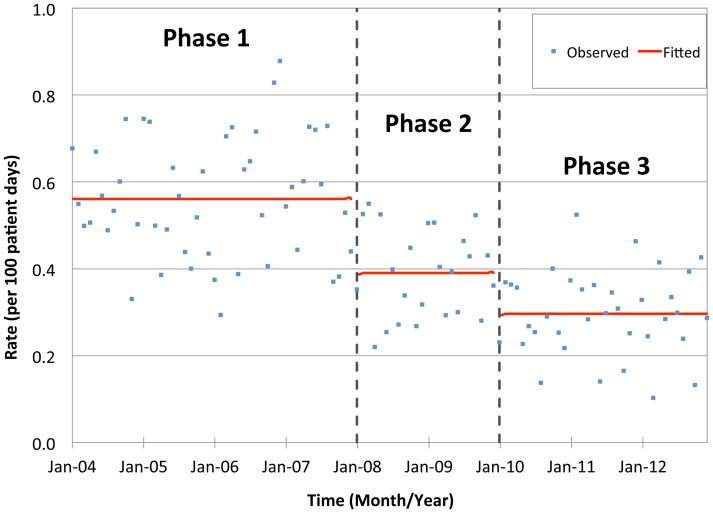
Incidence rate of hospital-acquired MRSA per 1000 patient-days in the three phases of intervention.

**Figure 3:**
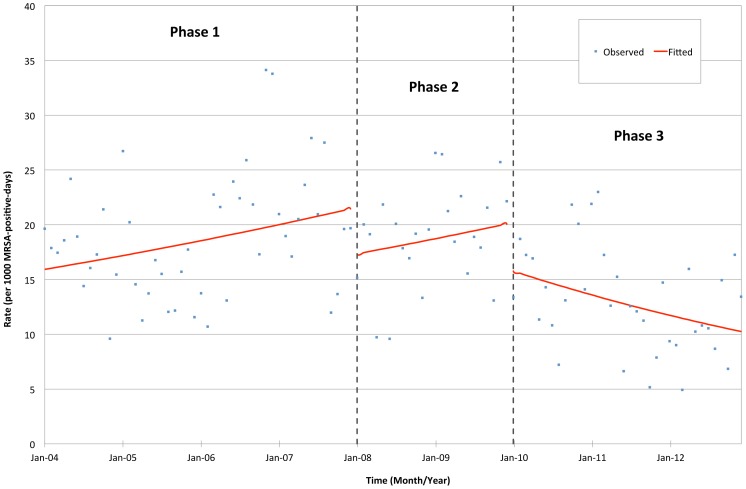
Incidence rate of hospital-acquired MRSA per 1000 MRSA-positive-days in three different phases of intervention.

The incidence rates of hospital-acquired MRSA per 1000 admissions and 1000 patient-days decreased significantly from phase 1 to 2 (p<0.001) and from phase 2 to 3 (p<0.001), equivalent to a cumulative reduction of about 50%; while there was insignificant trend within each phase. The incidence rate of hospital-acquired MRSA per 1000 MRSA-positive-days also reduced significantly from phase 1 to 2 (p = 0.040), and from phase 2 to 3 (p = 0.041); moreover, there was a significant increasing trend within phases 1 and 2 (p = 0.007), but a decreasing trend was noted within phase 3 (p = 0.001) (Table 2, Figures 1–3). On the other hand, the incidences rate of community-acquired MRSA per 1000 admissions remained unchanged throughout the study period.

**Table 2:** Changes in incidence of MRSA in three different phases of intervention.

	Immediate intervention impact				Pre- intervention trend		Change in trend	
	Phase 2 vs Phase 1		Phase 3 vs Phase 2				Phase 3 vs Phases 1 & 2^1^	
	IRR (95% CI)^2^;p-value	Percent change(95% CI)^3^	IRR (95% CI)^2^;p-value	Percent change(95% CI)^3^	IRR (95% CI)^2^;p-value	Percent change(95% CI)^3^	IRR (95% CI)^2^;p-value	Percent change(95% CI)^3^
Incidence rate of hospital-acquiredMRSA per 1000 admissions	0.637 (0.560, 0.724);p<0.001	−36.3%(−44.0%, −27.6%)	0.726 (0.625, 0.842);p<0.001	−27.4%(−37.5%, −15.8%)	NS	NS	NS	NS
Incidence rate of hospital-acquiredMRSA per 1000 patient-days	0.696 (0.612, 0.791);p<0.001	−30.4%(−38.8%, −20.9%)	0.759 (0.654, 0.881);p<0.001	−24.1%(−34.6%, −11.9%)	NS	NS	NS	NS
Incidence rate of hospital-acquiredMRSA per 1000 MRSA-positive-days	0.804 (0.652, 0.990);p = 0.040	−19.6%(−34.8%, −1.0%)	0.781 (0.615, 0.990);p = 0.041	−21.9%(−38.5%, −1.0%)	1.006 (1.002, 1.011);p = 0.007	0.6%(0.2%, 1.1%)	0.982 (0.971, 0.992);p = 0.001	−1.8%(−2.9%, −0.8%)

Note. Phase 1: baseline observation period; phase 2: launch of the first intervention - hand hygiene campaign; phase 3: continuation of phase 2 plus launch of the second intervention - contact precautions;

1 Insignificant change in trend in Phase 2 vs Phase 1 was found for all models, implying the same slope within Phases 1 and 2;

2 IRR denotes incidence rate ratio obtained from segmented Poisson regression, and CI denotes confidence intervals;

3 Percentage change in incidence rate obtained from (IRR−1)×100%; NS, not significant.

### Infection Control Program for MRSA

All patients with MRSA cultured from clinical specimens were assessed by the infection control team at bedside. Except for the 275 (6.5%) of 4256 MRSA-positive patients managed in the adult intensive care unit after July 2004 where isolation room was available after renovation [13], all other patients with MRSA colonization or infection were managed by standard precautions in the baseline period (phase 1). Hand hygiene using alcohol-based hand rub was actively promoted since 2008 (phase 2), during which hospital-wide hand hygiene compliance had increased from 23.2% (baseline in 2007), to 57.8% (2008), 67.5% (2009), 66.4% (2010), 76.3% (2011), and 78.6% (2012). In phase 3, contact precautions were implemented where a set of dedicated medical equipment were given to MRSA-positive patients who were otherwise nursed in open cubicles. In addition, MRSA-positive patients were bathed daily with 2% chlorhexidine gluconate. Throughout the study period, decolonization therapy with intra-nasal mupirocin was used in 88 (2.1%) specially selected MRSA-positive patients, out of a total of 4256, from 5 different clinical specialties. Incidence of nosocomial transmission of MRSA did not exceed 2 SD of the mean value in any of these wards. Thus no outbreak investigation was conducted during the study period. Environmental cleaning of clinical areas, especially frequently touched communal areas, such as bed rails and bed tables, were disinfected with 1000 ppm sodium hypochlorite at least once daily in all general wards. The usage density of broad-spectrum antibiotics with potential for selecting multiple-drug-resistant organisms, including cefepime, ceftazidime, cefotaxime, ceftriaxone, piperacillin, piperacillin/tazobactam, ticarcillin/clavulanate, cefoperazone/sulbactam, meropenem, imipenem/cilastatin, vancomycin, linezolid (iv/po), ciprofloxacin (iv/po), levofloxacin (iv/po), moxifloxacin (iv/po), ofloxacin (iv/po), in the clinical specialties of intensive care and high dependency unit, medicine, surgery, orthopedic, and oncology has increased steadily from 132.02 (phase 1), and 144.95 (phase 2), to 168.99 per 1,000 patient-days (phase 3).

## Discussion

The nosocomial transmission of MRSA in our hospital has been successfully reduced by half in terms of 1000-patient admission and 1000-patient-day during the study period. We have demonstrated that even without the use of isolation room in resource limited settings, nosocomial transmission of MRSA can be minimized by the promotion of hand hygiene using alcohol-based hand rub, implementation of contact precautions using dedicated medical equipment and use of 2% chlorhexidine gluconate for daily bathing when MRSA-positive patients were nursed in open cubicles. Contact precautions and isolation has been advocated for patients colonized or infected with MRSA, as recommended by Health Care Infection Control Practices Advisory Committee, US [18], [19]. However, isolation may be associated with less direct healthcare worker-patient contact time with which may potentially result in adverse outcomes [20], [21], especially for the critically ill patients. In fact, isolation of patients did not reduce nosocomial MRSA transmission in intensive care unit as illustrated in a prospective two-centre study [22], even though the unfavorable outcome of this study may be attributed to low compliance of hand hygiene. The role of patient isolation in the control of nosocomial MRSA transmission remains controversial [23], [24].

In our present study, implementation of hand hygiene campaign with an overall compliance of over 60% in phase 2 achieved a significant immediate reduction of incidence rate of hospital-acquired MRSA per 1000-admission, 1000 patient-days and 1000 MRSA-positive-days. Compellingly, the trend of hospital-acquired MRSA per 1000 MRSA-positive-days did not decrease until the introduction of contact precautions with dedicated medical equipment and the use of 2% chlorhexidine gluconate daily bathing in phase 3. This observation suggested that use of hand hygiene alone may not be sufficient in the control of MRSA nosocomial transmission in patients nursed in open cubicles.

Multifaceted or bundle approach was always adopted to control MRSA in hospitals [1], [25]. Although it is difficult to delineate the relative contribution of each component, it is important to identify the critical control points, which are the minimally required measures in resource limited settings. In phase 3, we have demonstrated that the use of contact precautions with dedicated medical equipment, along with 2% chlorhexidine gluconate daily bathing and hand hygiene practice further reduced hospital-acquired MRSA in our endemic setting. In fact, use of chlorhexidine gluconate daily bathing has been associated with a reduction of hospital-acquired infections, including MRSA, in previous studies [26], [27].

Antibiotic exposure was shown to be associated with MRSA acquisition [28]. During this study, despite the increasing trend in broad-spectrum antibiotics consumption, which was predictable in view of the importation of multiple-drug resistant organisms in our community [29], [30], [31], appropriate infection control measures as described had seemingly overcome the antibiotic selective pressure in the control of MRSA nosocomial transmission [1], [13]. However, the consumption of antibiotics with activity against MRSA such as vancomycin and linezolid were also increased from phase 1 to 3. We postulated that this was related to the frontline clinician’s choice of empirical antibiotic treatment. As there was a high incidence of MRSA in phase 1, the clinicians might have adopted the use of these antibiotics as their empirical treatment regimen since phase 1 which extended into phases 2 and 3. As there might be a lag time period before they would revert their empirical treatment regimen back to agents without activity against MRSA until they noticed a steady decrease in the prevelance of MRSA subsequently, the consumption of vancomycin and linezolid continued to rise in phases 2 and 3. Further long-term follow-up studies should be conducted to assess this hypothesis.

There are several limitations in this study. Firstly, we did not perform universal admission screening for MRSA prospectively due to resource constraint, hence, the true incidences of community-acquired and hospital-acquired MRSA could not be determined. Our data presented might represent an underestimation in the incidence of community-acquired MRSA and an overestimation in the incidence of hospital-acquired MRSA. However, this potential overestimation in the incidence of hospital-acquired MRSA has remained constant throughout the study period and would, therefore, be unlikely to have affected the extent of the reduction in incidence after the implementation of our infection control measures. Secondly, we introduced 2% chlorhexidine gluconate daily bathing in the later stage of phase 3 so that the relative contribution of each component in phase 3 could not be clearly illustrated. However, we believe that our infection control program can serve as an example for resource limited settings where active surveillance culture and isolation facilities are lacking. Finally, the increasing consumption of antibiotics with activity against MRSA throughout the study period might have constituted a confounding factor by decreasing the nosocomial incidence of MRSA. However, since other broad spectrum antibiotics which had selective pressure on MRSA also increased, the stepwise decrease in the incidence of MRSA from phase 1 to 3 were likely related to the infection control interventions applied in phases 2 and 3 in our resource limited setting, though this potential confounding factor should be further evaluated in future studies.
